# Supplementary material for: Integrative analysis of single-cell RNA-seq and gut microbiome metabarcoding data elucidates macrophage dysfunction in mice with DSS-induced ulcerative colitis
Source: Commun Biol. 2024 Jun 15;7:731. doi: 10.1038/s42003-024-06409-w (PMC11180211; doi:10.1038/s42003-024-06409-w)
Supplement: Supplementary file 2 — Supplementary Information [file 42003_2024_6409_MOESM2_ESM.pdf]

Supplementary Information on

Integrative analysis of single-cell RNA-seq and gut microbiome metabarcoding data elucidates macrophage dysfunction in mice with DSS-induced ulcerative colitis

Dawon Hong[1†], Hyo Keun Kim[2†], Wonhee Yang[3], Chanjin Yoon[4], Minsoo Kim[5], Chul-Su Yang[6*], Seokhyun Yoon[7*]

Assessing the colitis score

For clinical score of colitis, Body weight, occult or gross blood lost per rectum, and stool consistency were determined every other day during the colitis induction based on the scoring system shown in Supplementary Table S1 (Clin Microbiol Rev. 2002;15:79–94; Dig Dis Sci. 1993;38:1722-34). Weight loss was defined as the difference between initial and final weights, and diarrhea as the absence of fecal pellet formation and the presence of continuous fluid fecal material in the colon. Rectal bleeding was assessed based on the presence of diarrhea containing visible blood and on the presence of gross rectal bleeding. Clinical score of colitis values were calculated as ((weight loss score) + (diarrhea score) + (rectal bleeding score))/4. The clinical score was assessed by three trained investigators blinded to the treatment groups who were not aware of the treatment.

Supplementary Table S1. Criteria for disease activity index

Score	Weight loss (%)	Stool Consistency	Bloodstain or gross Bleeding
0	None	Normal	Negative
1	1-5	Loose stool	Negative
2	5-10	Loose stool	Positive
3	10-15	Diarrhea	Positive
4	>15	Diarrhea	Gross bleeding

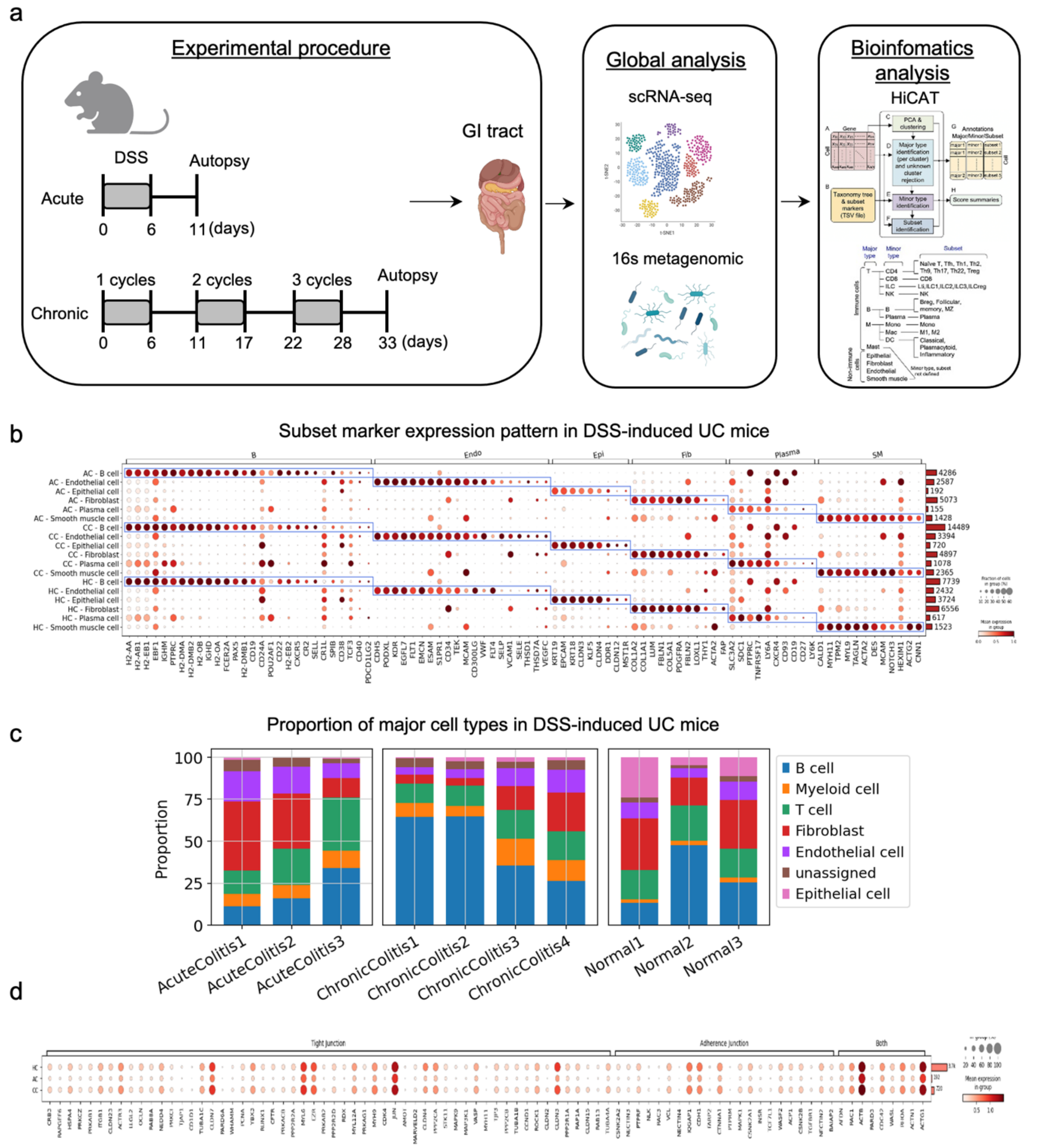

Supplementary Figure S1. Epithelial cell profiling in single-cell RNA-seq in DSS-induced mouse colitis. **a**: Overview of the workflow, **b**: Subset marker expression pattern. **c**: Bar plot showing cell type (major type) proportion per sample. **d**: Dot plot depicting the expression profiles of cell barrier-related genes in epithelial cell population.

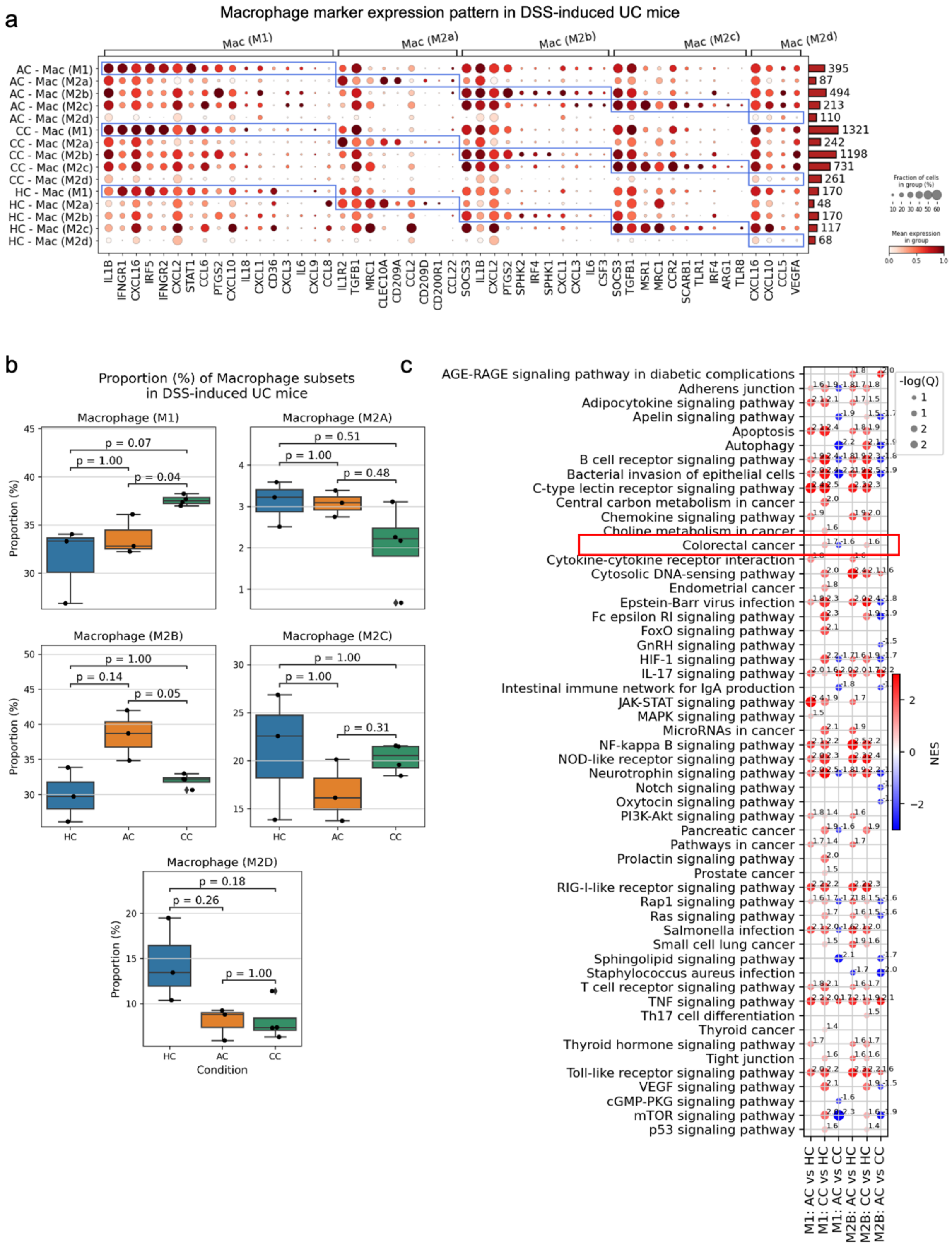

Supplementary Figure S2. Macrophage profiling in single-cell RNA-seq in DSS-induced UC mice. **a**: Macrophage subset marker expression pattern per condition. **b**: Bar plot showing subset proportion of macrophage in different condition. Subset proportion (%) were measured for each sample (n = 3, 3 and 4 for HC, AC and CC, respectively) **c**: Dot plot showing the gene set enrichment analysis (GSEA) results for M1 and M2B macrophages. We used DEGs for M1 and M2B comparing AC versus HC, CC versus HC and AC versus CC, respectively. Dot size corresponds to the negated log of adjusted p-value while color to normalized enrichment score.

Chronic activation of NLRP3 inflammasome

Our main points in [5] were as follows. (Supplementary Figure S3A)

1) Increased interaction between Visfatin (eNAMPT) and NOX2 complex (consisting of CYBB, CYBA, NCF1/2/4) increases the ROS level in macrophages.
2) Increased ROS level activate NLRP3 inflammasome to cleaves pro-IL1β to mature IL-1β. (Activation signaling)
3) eNAMPT-Tlr4 interaction induce NF-κB-mediated NLRP3 and pro-IL-1β expression. (Priming signaling)

To check if they really happen in DSS-induced UC mice, specifically in chronic UC, we first checked the expression of *Nampt* and of genes comprising the NOX2-complex, including *Cybb*, *Cyba*, *Ncf1*, *2*, and *4* in macrophages (Figure 4B and Supplementary Figure S3B). As expected from our previous findings, they were mostly over-expressed significantly, except *Nampt* and *Ncf1*. However, even with the insignificance of *Nampt* and *Ncf1*, the tendencies look similar to those of other genes. Increased ROS (reactive oxygen species) level can be implicitly confirmed from the over-expression of ROS related genes, such as *Sod2*, *Neat1*, and *Hif1a* (Supplementary Figure S3C). The genes involved in priming of NLRP3 inflammasome, such as *Tlr4*, *Nfkb1*, and *Nlrp3* were also significantly over-expressed in CC when compared to HC (Figure 4E). Of note, the increased expression level does not necessarily mean only the increased production rate in individual macrophage, but also the increase in the fraction of macrophages expressing these genes, i.e., alternately polarized macrophages. The violin plots in Supplementary Figure S3E clearly shows the higher fraction of macrophages are expressing those genes around NLRP3 inflammasome in chronic colitis than those in healthy colon.

Certainly, the increase in the number of RNAs does not necessarily mean the increase in protein level, e.g., the over-expression of IL1β gene in RNA level, which is actually pro-IL1β, does not necessarily mean higher level of IL-1β secretion. Therefore, we measured protein level expression of those genes from both colon lysates (Figure 4C) and colon macrophages (Figure 4D) separately. In both cases, the tendencies we found from RNA level could be confirmed in protein level too.

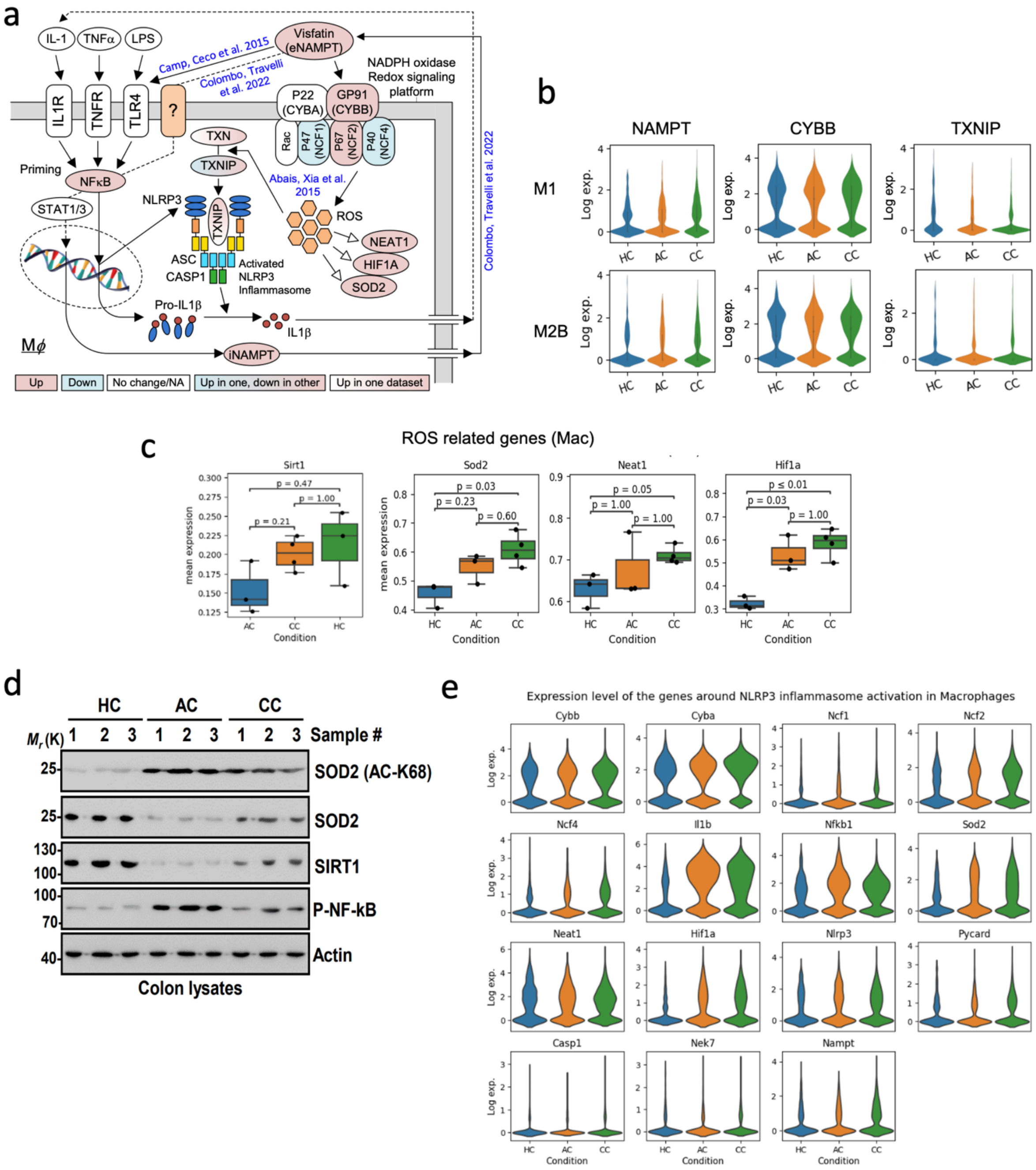

Supplementary Figure S3. Expression profiling of the NLRP3 inflammasome activation. **a**: Schematic diagram of *Nampt*'s binding with *Tlr4* and NOX2 complex-mediated NLRP3 inflammasome activation. **b**: Violin plots for the expression of *Nampt*, *Cybb*, and *Txnip* in macrophages. **c**: Box plots showing the per-sample mean expression of ROS-related genes in macrophages. The mean expression levels were measured for each sample (n = 3, 3 and 4 for HC, AC and CC, respectively) **d**: Western blotting result showing the protein level expression of ROS-related genes. **e**: Violin plots showing the expression of genes associated with NLRP3 inflammasome activation.

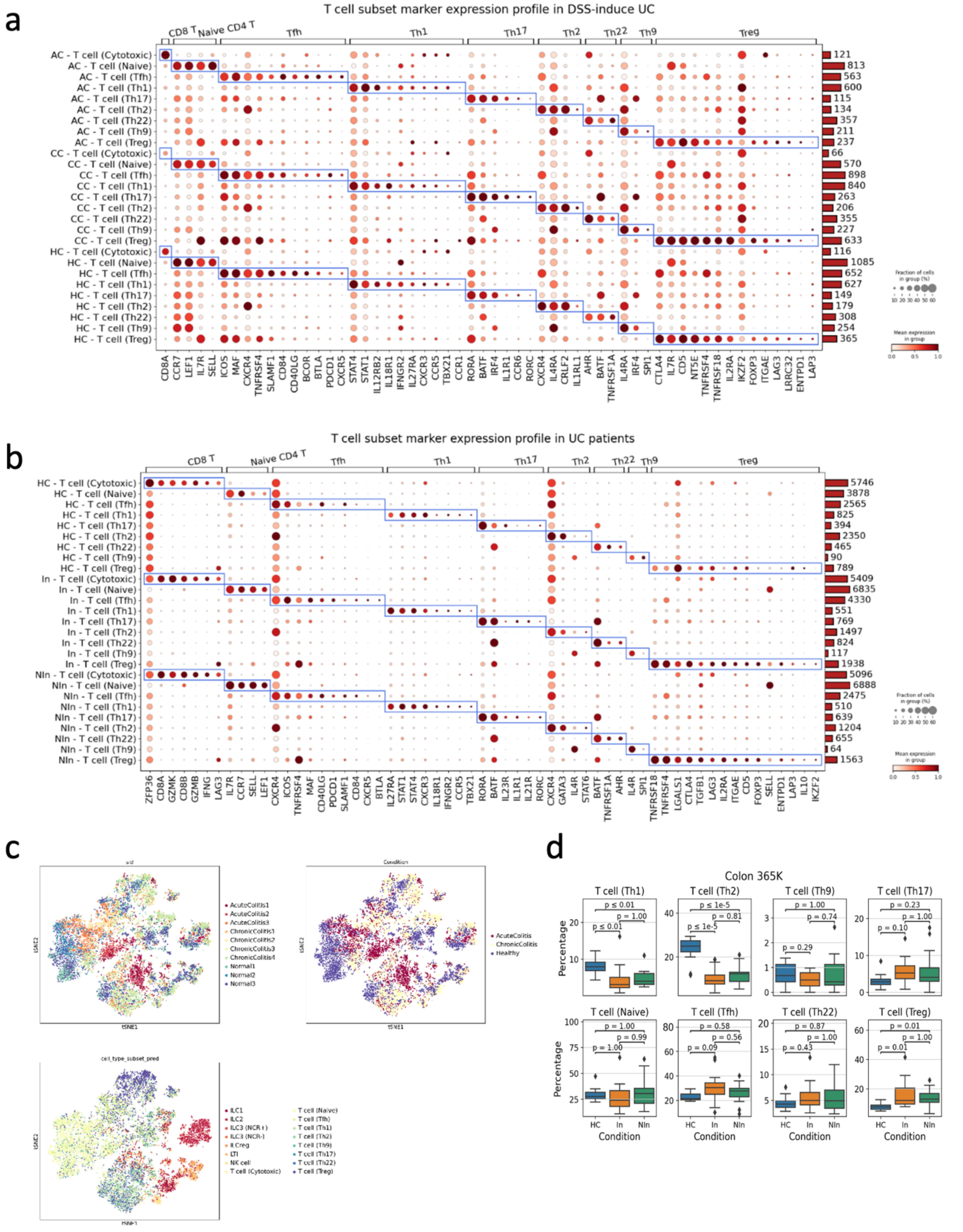

Supplementary Figure S4. T cell profiling in single-cell RNA-seq in DSS-induced UC mice. **a**: T cell subset marker expression pattern per condition in UC mice. **b**: T cell subset marker expression pattern per condition in human UC patients (SCP259 dataset). **c**: tSNE plot for T cell clusters in DSS-induced UC mice. **d**: Bar plot showing T cell subset proportion in human UC patients (SCP259 dataset). Subset proportion (%) were measured for each sample and their distributions (box plots) for each condition were compared to each other. The p-values were obtained using t-test for each pair of conditions.

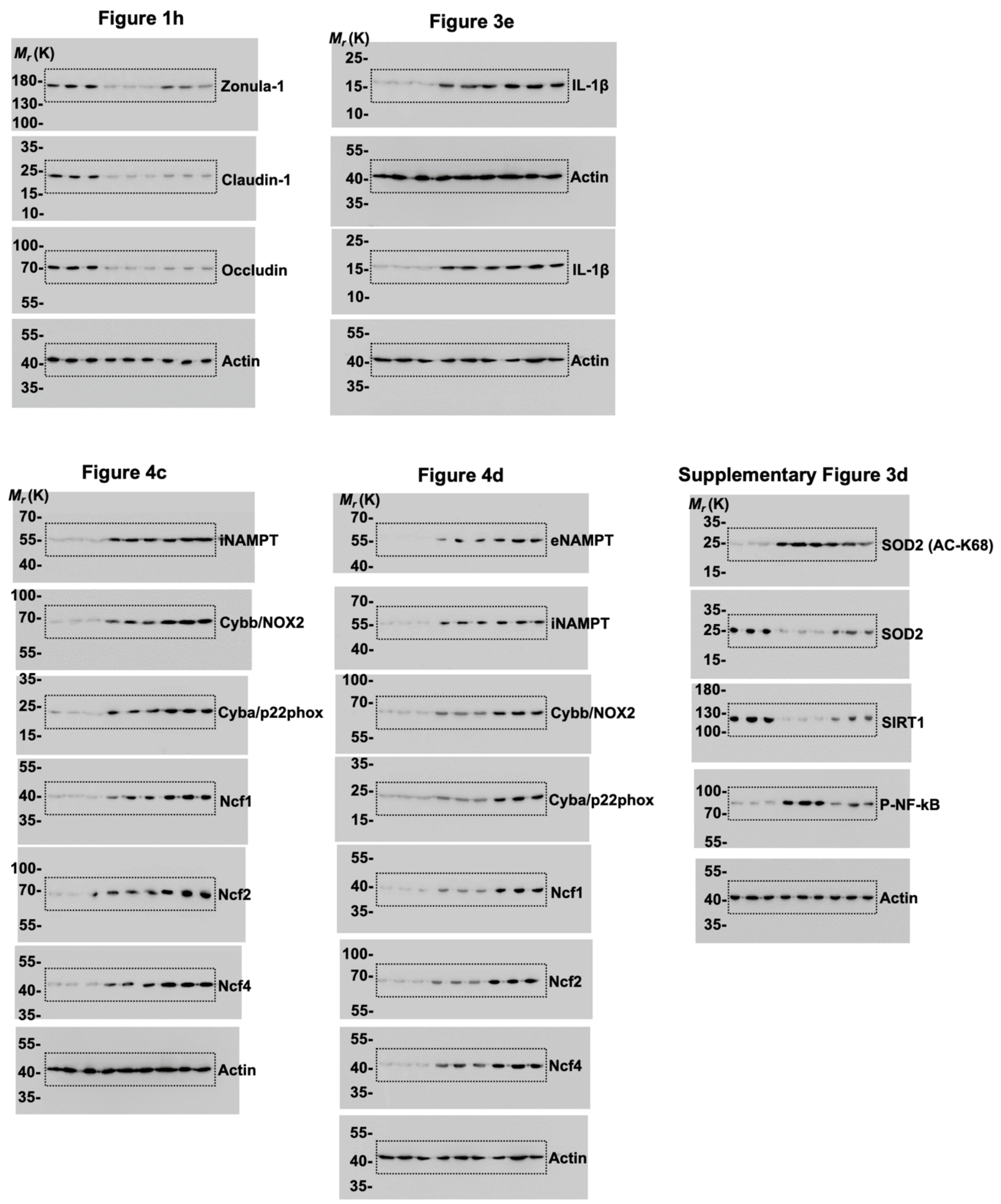

Supplementary Figure S5. Uncropped images for all the Western blots reported in this study.
