# Supplementary material for: Integrative analysis of single-cell RNA-seq and gut microbiome metabarcoding data elucidates macrophage dysfunction in mice with DSS-induced ulcerative colitis
Source: Commun Biol. 2024 Jun 15;7:731. doi: 10.1038/s42003-024-06409-w (PMC11180211; doi:10.1038/s42003-024-06409-w)
Supplement: Supplementary file 3 — Description of Additional Supplementary Materials [file 42003_2024_6409_MOESM3_ESM.pdf]

Description of Additional Supplementary Files

File name: Supplementary Data 1

Description: Ligand-receptor interactions found in Acute Colitis

File name: Supplementary Data 2

Description: Cell-type markers used. They were mostly obtained from R&D systems, https://www.rndsystems.com/resources/cell-markers

File name: Supplementary Data 3

Description: Percentage difference of microbiota in Family. Acute colitis versus Healthy

File name: Supplementary Data 4

Description: Percentage of microbiota in Family

File name: Supplementary Data 5

Description: ASV_table shared with tax assignment
